# Editorial Expression of Concern: Functional connectivity signatures of political ideology

**DOI:** 10.1093/pnasnexus/pgae569

**Published:** 2025-01-09

*PNAS Nexus* is publishing an Editorial Expression of Concern regarding the following article: Seo Eun Yang, James D Wilson, Zhong-Lin Lu, Skyler Cranmer, Functional connectivity signatures of political ideology, *PNAS Nexus*, Volume 1, Issue 3, July 2022, pgac066, https://doi.org/10.1093/pnasnexus/pgac066.

*Following publication of the article, a reader discovered a coding error which selected the hyperparameters by assessing their performance on a test dataset instead of the training dataset, as intended. The journal editors are posting this Editorial Expression of Concern while awaiting additional information from the authors, which will help determine what further action is required.*

