# Correction to: Functional connectivity signatures of political ideology

**DOI:** 10.1093/pnasnexus/pgaf212

**Published:** 2025-07-16

This is a correction to: Seo Eun Yang, James D Wilson, Zhong-Lin Lu, Skyler Cranmer, Functional connectivity signatures of political ideology, *PNAS Nexus*, Volume 1, Issue 3, July 2022, pgac066, https://doi.org/10.1093/pnasnexus/pgac066.

After publication, a reader alerted us to an error in our original code and a needed clarification of our cross-validation approach. We have corrected the errors and discuss their implications below. After a re-analysis, we found that the result of our study - that some functional connectivity tasks are highly correlated to and predictive of political ideology - remains unaltered when the code has been corrected.

Hyperparameter Tuning Error Corrected: Revised Results

Our article had an error in hyperparameter turning. Initially, we had mistakenly tuned hyperparameters for our Brain-Net CNN architecture using the test set in each fold of the cross validation. We have corrected this error in tuning, while keeping the model, architecture, and implementation the same as originally published. We have updated the publicly available code on Github (https://github.com/jdwilson4/ThePoliticalBrain) to reflect these changes. After fixing this error, we updated Fig. 1 and Fig. 3.

**Fig. 1.**
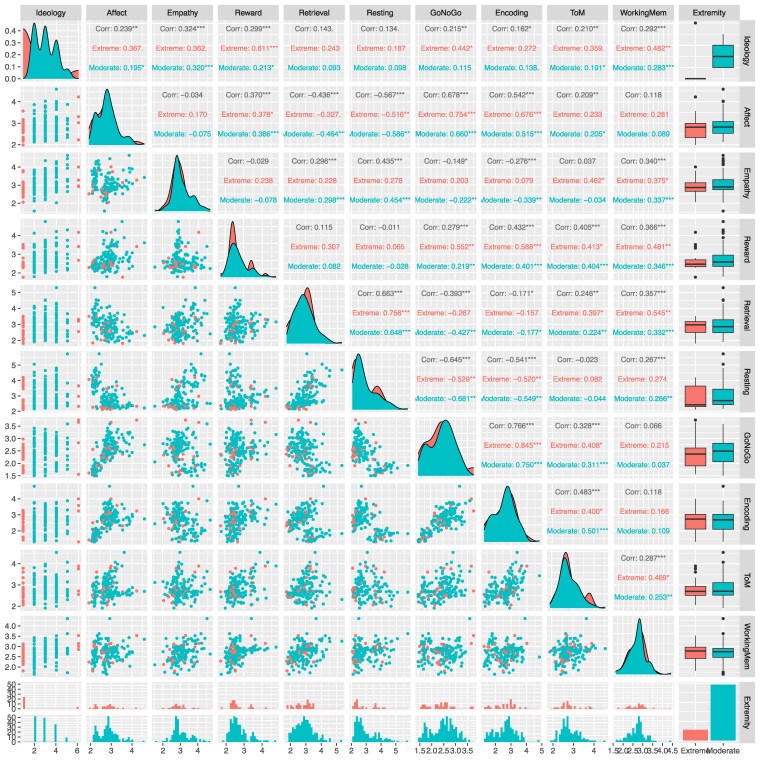
Pairwise associations of FC scores and their associations with political ideology. Scatterplots show the relationship between the predicted political ideology score from each FC task once applied to BrainNetCNN and the true ideology. Points are colored according to extremity of true ideology: red points show extreme views (very liberal or very conservative) and blue points show moderate views (liberal, somewhat liberal, moderate, somewhat conservative, and conservative). Correlations are provided for all ideology values (in black), moderate only (in blue), and extreme values (in red). Correlation values with *** are statistically significant with P-value < 0.001, those with ** are statistically significant with P-value < 0.01, and those with * are significant at P-value < 0.05.

**Fig. 3.**
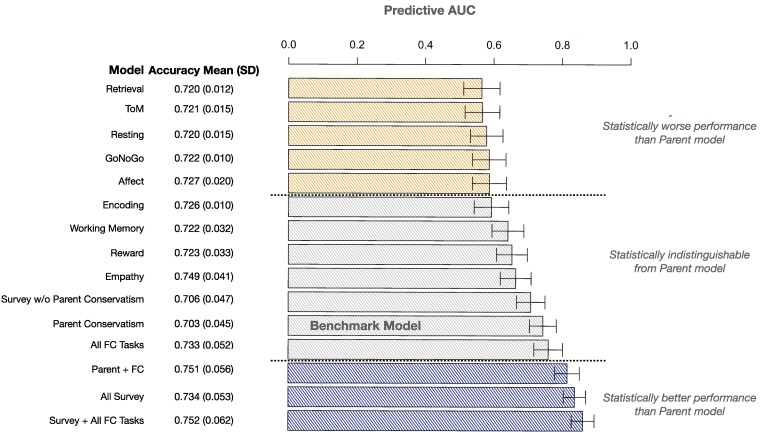
Prediction performance of FC and survey-based predictive models. Accuracy and AUC metrics were calculated for each model using Monte Carlo cross-validation, where the test set was a random sample with a random proportion of observations over 1,000 samples. The length of each bar in the plot represents the mean predictive AUC, and error bars represent 95% CIs. Bars are colored according to their performance when compared to the Parent Conservatism benchmark model containing mother and father conservatism as predictors. Survey-based models included age, education, income, how conservative the town was where a subject grew up, how conservative the city is where the subject lives now, a subject's parents’ income, and mother and father's conservatism.

The Reward task and the Empathy task remain the most strongly correlated with ideology and they are statistically correlated with moderate and extreme ideologies (See Table 1). After re-analysis and Bonferroni correction for multiple comparisons over nine tasks, we reaffirm the statistical significance of six of these key tasks at significance level 0.05 — Empathy, Reward, Working Memory, Affect, GoNogo, and ToM (See Table 1) — highlighting their predictive relevance in linking functional connectivity with political ideology.

**Table 1.** Out-of-sample evaluation for each task. We compute Pearson correlation coefficients between the predicted values and self-identified political ideology scores in the test dataset as well as their statistical significance (P-value). They are rounded up to four decimal places.

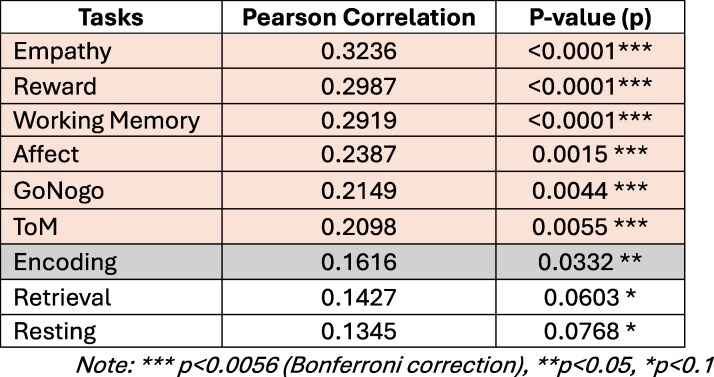

The comparisons of predictive accuracies and AUCs in Fig. 3 for the corrected analysis against the original analysis are similar to the findings of Fig. 1. The Retrieval task is no longer considered statistically indistinguishable in prediction performance from the Parent benchmark model. However, the number of FC tasks that are considered statistically indistinguishable in predictive performance to the Parent model is consistent with the original analysis (4 individual tasks + all tasks combined). Furthermore, the Reward and Empathy tasks are the most accurate and have the highest AUC among individual tasks like our findings in the original paper. Finally, the re-analysis upholds our finding that the addition of FC tasks to the parent conservatism data statistically improves the prediction of political ideology.

Clarification of Cross-Validation Approach

We would like to clarify a typographical error: our analysis used 4-fold cross-validation, not 10-fold cross-validation as written. Additionally, our approach differs from what is often seen as a standard cross-validation framework. In each fold, hyperparameters were re-optimized rather than being held constant across folds. That is, for every split, we selected hyperparameters based on a separate validation set within that fold and then evaluated performance on the corresponding test set. In this way, our approach is closer to conducting four separate validation set analyses, resulting in four models (with potentially different hyperparameters) and four predicted scores for the corresponding test sets. We then concatenate the predicted political ideology scores from all four test sets and compute the Pearson correlation between these aggregated predictions and the corresponding self-identified political ideology scores.

