# Supplementary material for: Functional connectivity signatures of political ideology
Source: PNAS Nexus. 2022 May 23;1(3):pgac066. doi: 10.1093/pnasnexus/pgac066 (PMC9291242; doi:10.1093/pnasnexus/pgac066)
Supplement: pgac066_Supplemental_Files [file pgac066_supplemental_files.zip › PNASNEXUS-PNASNEXUS-2021-00196-s02.pdf]

Supplemental Material for "Functional Connectivity Signatures of Political Ideology"

Seo Eun Yang[1], James D. Wilson[2*], Zhong-Lin Lu[3], and Skyler J. Cranmer[1]

[1]Department of Political Science, The Ohio State University, Columbus OH 43210

[2] Department of Psychiatry, University of Pittsburgh School of Medicine, Pittsburgh, PA 15213

[3] Center for Neural Science and Department of Psychology, New York University, New York, NY, 1000[illegible]

[*]To whom correspondence should be addressed. E-mail: wilsonj41@upmc.edu

Materials and Methods

We use brain imaging data collected from 174 participants at the Ohio State University and its vicinity (age 18-40, mean 21.4; 61 males and 113 females). After preprocessing the data following the Human Connectome Project pipeline, brain images were parceled into 269 regions of interest (ROIs) using the Automated Anatomical Labeling atlas. We excluded the 252th ROI because it had all missing values in the time series of averaged BOLD signals, resulting in 268 x 268 symmetric matrix. Details of data acquisition are in appendix A. We recorded BOLD functional activation when subjects were in resting state and performed eight emotional and cognitive tasks. The task associated BOLD activations were regressed out from the time series before connectivity analysis (?). We construct functional connectivity networks by creating matrices where each row and column represent an ROI and the value of the (i, j) entry of the matrix is the correlation coefficient between the i-th and j-th brain region from the time series of averaged BOLD signals. We create such matrices for each participant and each task so that there are

nine matrices per participant, one matrix for each of the nine tasks. Each task-specific functional connectivity matrix is represented as a full symmetric matrix with zeros along the diagonal and is between [-1,1].

The outcome measure we consider is a subject's self-reported ideological position on a six point Likert scale from "Very liberal" to "Very conservative." We consider the outcome measure continuous values and predict the actual ideological scores directly through a deep learning approach because having an actual predicted score would be more informative. Participants were also provided a series of survey-based questions, including questions regarding age, gender, their education and income, the education and income of their parents, the conservatism of their parents, as well as the conservatism of the city that they grew up in and the city they now live. With the exception of age and gender, these survey questions were answered on Likert scales. We use these covariates to build predictive models for political ideology as a benchmark against which we assess the utility of functional connectivity for predicting ideology. We provide descriptive summaries of these variables in Table **??**, including the correlation of each feature with the Likert scale value of political ideology. We provide the survey questions and their possible answers in supplementary material Section S1.

While previous neuropolitical studies have typically employed a single task designed to elicit responses related to political orientation, the subjects in our data are scanned while performing a series of eight tasks, as well as in the resting state. None of these tasks are designed to elicit partisan responses and the resting state scan is particularly interesting because it allows us to test if an internal brain connectivity predict a subject's political orientation without any stimulus at all. Further, examining the resting state as well as these eight distinct tasks helps provide a general overview for the predictive ability of all tasks that distinctively capture brain regions related to political ideology. The eight tasks

aim to observe subjects' brain activity involved in emotional picture viewing, emotional face viewing, episodic memory encoding and episodic memory retrieval, Go/No-go, monetary incentive, working memory, and a theory of mind task (see Supplementary Table S1 for task descriptions).

Data acquisition

MRI recording was performed using a standard 12-channel head coil on a Siemens 3T Trio Magnetic Resonance Imaging System with TIM, housed in the Center for Cognitive and Behavioral Brain Imaging at the Ohio State University (OSU). BOLD functional activations for tasks were measured with a T2*-weighted echo-planar image sequence (repetition time= 2000 ms, echo time= 28 ms, flip angle = 72 deg, field of view = 222 x 222, in-plane resolution = 74 $\cdot$ 74 pixels or 3mm x 3mm, 38 slices with thickness of 3mm). The resting-state acquisition had higher resolution (repetition time= 2500 ms, echo time = 28 ms, flip angle = 75 deg, in-plane resolution = 2.5mm x 2.5mm, 44 slices with thickness of 2.5 mm). T1-weighted structural images were acquired for each subject with the three-dimensional magnetization-prepared 180 radio frequency pulses and rapid gradient-echo (MPRAGE) sequence (1 x 1 x1mm^3 resolution, inversion time = 950ms, repetition time = 1950 ms, echo time = 4.44 ms, flip angle = 12 deg, matrix size= 256 x 224, 176 sagittal slices per slab; scan time 7.5min). Stimuli were presented to subjects on a rear projection screen through a mirror on top of the head coil. Visual stimuli were generated on a Windows computer running MATLAB programs based on Psychtoolbox extensions. During the 1.5-h MRI session, each subject performed eight behavioral tasks designed to target basic cognitive functions: emotional picture viewing (360 sec), emotional face viewing (360 sec), episodic memory encoding (304 sec), episodic memory retrieval (252 sec), Go/No-go (360 sec), monetary incentive (456 sec), working memory

(354 sec), and theory of mind stories/questions (376 sec). Resting-state scans were also recorded for each subject (360 sec). Task descriptions are presented in Supplementary Table S3. For convenience of description, resting state is treated as one of the nine tasks.

S1. Survey Questions Analyzed in the Study

The survey questions from the Wellbeing study at the Ohio State University that were analyzed as demographic and socio-economic controls in the primary study.

Q7 Are you male or female?

- ❍ Male
- ❍ Female

Q9 What is your age?

Q17 How religious are you?

- ❍ Not at all
- ❍
- ❍
- ❍
- ❍
- ❍ Very religious

Q25 What is the highest level of education for you and your parents?

	High school or lower	Some college	College graduate	Post-college education	Graduate or professional degree
You					
Your father					
Your mother					

Q26 How liberal or conservative do you rate yourself and your parents?

	Very liberal					Very conservative
You						
Your father						
Your mother						

Q28 What is the yearly household income for you and your parents?

	< $30,000	$30,000-$50,000	$50,001-$70,000	$70,001-$90,000	$90,001-$120,000	>$120,000
You						
Your parents						

Q31 How liberal or conservative was the town or city where you grew up?

- ❍ Very liberal
- ❍
- ❍
- ❍
- ❍
- ❍ Very conservative

Q34 How liberal or conservative is the town or city where you live now?

- ❍ Very liberal
- ❍
- ❍
- ❍
- ❍
- ❍ Very conservative

Table S2. Anatomical structures in the AAL atlas

We follow a list of anatomical structures in the AAL atlas illustrated in (?). Only the 78 structures were included, cerebellum was not considered. Here, the full names as well as their short abbreviation used in some figures are specified.

**Brain Lobe**	**Short Name**	**Full Name**	**Corresponding ROIs number**
Frontal	SFGdor-R	Right Superior frontal gyrus, dorsolateral	184,185,189, 191,192,193, 196,198,200
Frontal	SFGdor-L	Left Superior frontal gyrus, dorsolateral	82,96,105
Frontal	MFG-R	Right Middle frontal gyrus	213,215,224, 225,237
Frontal	MFG-L	Left Middle frontal gyrus	35,41,51, 56,57,66, 68,69,73, 80,81
Frontal	ORBmid-R	Right Middle frontal gyrus, orbital part	202,227
Frontal	ORBmid-L	Left Middle frontal gyrus, orbital part	39
Frontal	IFGoperc-R	Right Inferior frontal gyrus, opercular part	228,252
Frontal	IFGoperc-L	Left Inferior frontal gyrus, opercular part	11,46

Table S3. Task descriptions

Brain Lobe	Short Name	Full Name	Corresponding ROIs number
Frontal	IFGtriang-R	Right Inferior frontal gyrus, triangular part	241,248
Frontal	IFGtriang-L	Left Inferior frontal gyrus, triangular part	17,26,31
Frontal	ORBinf-R	Right Inferior frontal gyrus, orbital part	216,239
Frontal	ORBinf-L	Left Inferior frontal gyrus, orbital part	30,48,61
Frontal	SMA-R	Right Supplementary motor area	156,165,168
Frontal	SMA-L	Left Supplementary motor area	102,113,121,133
Frontal	SFGmed-R	Right Superior frontal gyrus, medial	145,148,161, 163,164
Frontal	SFGmed-L	Left Superior frontal gyrus, medial	109,111,129, 130,132
Frontal	ORBsupmed-R	Right Superior frontal gyrus, medial orbital	146
Frontal	ORBsupmed-L	Left Superior frontal gyrus, medial orbital	123,125
Frontal	PCL-R	Right Paracentral lobule	154
Frontal	PCL-L	Left Paracentral lobule	100,104,134
Insula	INS-R	Right Insula	201,214,219, 223,233,243
Insula	INS-L	Left Insula	55,63
Parietal	SPG-R	Right Superior parietal gyrus	182,187
Parietal	SPG-L	Left Superior parietal gyrus	76,90
Parietal	IPL-R	Right Inferior parietal, but supramarginal and angular gyri	212,244,256
Parietal	IPL-L	Left Inferior parietal, but supramarginal and angular gyri	14,18,53,71
Parietal	SMG-R	Right Supramarginal gyrus	221,260,267
Parietal	SMG-L	Left Supramarginal gyrus	2,4
Parietal	ANG-R	Right Angular gyrus	230,251
Parietal	ANG-L	Left Angular gyrus	13,32,34
Parietal	PCUN-R	Right Precuneus	147,149,150, 159,174
Parietal	PCUN-L	Left Precuneus	107,110,112, 136,137
Central	PreCG-R	Right Precentral gyrus	220,231,235, 254,255,265
Central	PreCG-L	Left Precentral gyrus	6,21,33,43,74
Central	ROL-R	Right Rolandic operculum	232,245,261,266
Central	ROL-L	Left Rolandic operculum	3,12,37,44
Central	PoCG-R	Right Postcentral gyrus	197,210,222,257
Central	PoCG-L	Left Postcentral gyrus	27,38,49,60,72
Limbic	ACG-R	Right Anterior cingulate and paracingulate gyri	155
Limbic	ACG-L	Left Anterior cingulate and paracingulate gyri	114,116,119,127
Limbic	DCG-R	Right Median cingulate and paracingulate gyri	151,152,153,160
Limbic	DCG-L	Left Median cingulate and paracingulate gyri	103,115,124, 128,135
Limbic	PCG-L	Left Posterior cingulate gyrus	139
Limbic	HIP-R	Right Hippocampus	199,218
Limbic	HIP-L	Left Hippocampus	78
Limbic	PHG-R	Right Parahippocampal gyrus	195,205
Limbic	PHG-L	Left Parahippocampal gyrus	67,85,97
Limbic	TPOsup-R	Right Temporal pole: superior temporal gyrus	229,253
Limbic	TPOsup-L	Left Temporal pole: superior temporal gyrus	22,40
Limbic	TPOmid-R	Right Temporal pole: middle temporal gyrus	240

Brain Lobe	Short Name	Full Name	Corresponding ROIs number
Temporal	STG-R	Right Superior temporal gyrus	250,258,259
Temporal	STG-L	Left Superior temporal gyrus	5,9,19,36,42
Temporal	MTG-R	Right Middle temporal gyrus	246,249,262, 263,264,269
Temporal	MTG-L	Left Middle temporal gyrus	1,7,8,10,15, 16,20,23,24
Temporal	ITG-R	Right Inferior temporal gyrus	242
Temporal	ITG-L	Left Inferior temporal gyrus	28,29,50
Occipital	CAL-R	Right Calcarine fissure and surrounding cortex	158,162,173,181
Occipital	CAL-L	Left Calcarine fissure and surrounding cortex	86,99,117,143
Occipital	CUN-R	Right Cuneus	178
Occipital	CUN-L	Left Cuneus	92,101,144
Occipital	LING-R	Right Lingual gyrus	167,170,177,194
Occipital	LING-L	Left Lingual gyrus	84,93,108
Occipital	SOG-R	Right Superior occipital gyrus	186,203
Occipital	SOG-L	Left Superior occipital gyrus	95
Occipital	MOG-R	Right Middle occipital gyrus	208,211,236
Occipital	MOG-L	Left Middle occipital gyrus	45,54,64,65,77
Occipital	IOG-R	Right Inferior occipital gyrus	209,234
Occipital	IOG-L	Left Inferior occipital gyrus	47
Occipital	FFG-R	Right Fusiform gyrus	188,206,217,238
Occipital	FFG-L	Left Fusiform gyrus	58,75,87
Subcortical	AMYG-R	Right Amygdala	207
Subcortical	AMYG-L	Left Amygdala	83
Subcortical	CAU-R	Right Caudate nucleus	169,172
Subcortical	CAU-L	Left Caudate nucleus	88,91,106
Subcortical	PUT-R	Right Lenticular nucleus, putamen	190,204
Subcortical	PUT-L	Left Lenticular nucleus, putamen	62,70,79
Subcortical	THA-R	Right Thalamus	157,175
Subcortical	THA-L	Left Thalamus	89,98,118

**Task**	**Description**
Affect (Emotional pictures)	Subjects see photographs of the screen, one at a time. These photographs appear to the left or right of the center of the screen. The task is to indicate whether the picture is shifted to the left or right relative to green dot in the center of the screen. (?)
Empathy (Emotional faces)	Subjects are presented with male and female faces, one at a time. The task is to determine whether the faces are male or female. There are task conditions for neutral, happy, sad, and fearful faces (?).
Episodic Memory (Encoding)	Subjects see name and face pairings on a screen. The task is to decide whether the name goes well with the face on a 1-4 (poor to well) scale. There are 4 face conditions: young and old faces that are novel or have been repeated during the experiment (?).
Episodic Memory (Retrieval)	Subjects are asked to remember which names were paired with which faces from the episodic memory encoding task. The task is to indicate whether the face name pairs are the same from the previous task, completely novel, or if the face is repeated, but was not paired with the given name (?).
Go/No-Go	Subjects look images of single letters. They are asked to press a button when the letter is in the set A,B,C,D,E and not to press the button when the letter is in the set X,Y,Z (?).
Reward (Monetary Incentive Delay)	Subjects are asked to press a button as quickly as possible when a white square (cue) appears on the screen. Participants either win or lose money based on when and how fast they push the button (?).
Working Memory	Subjects are presented with a sequence of letters and switch between two memory tasks. In the rst, subjects are asked to indicate whether the current letter is underlined. In the second, subjects are asked to indicate whether the current letter is the same as or dierent from the one that was presented two letters ago (?).
Theory of Mind (ToM)	Subjects are presented with stories and true false statements about the stories. The task is to indicate whether the statement was true or false (?).
Resting State	Subjects are asked to close eyes, feel relaxed but stay awake.
