# Supplementary material for: Functional connectivity signatures of political ideology
Source: PNAS Nexus. 2022 May 23;1(3):pgac066. doi: 10.1093/pnasnexus/pgac066 (PMC9291242; doi:10.1093/pnasnexus/pgac066)
Supplement: pgac066_Supplemental_Files [file pgac066_supplemental_files.zip › PNASNEXUS-PNASNEXUS-2021-00196-s01.pdf]

PNAS Nexus, 2021, pp. 1–12

doi: DOI HERE
Advance Access Publication Date: Day Month Year
Paper

PAPER

Functional Connectivity Signatures of Political Ideology

Seo-Eun Yang,[a, 1] James D. Wilson,[b, 1, *] Zhong-Lin Lu[c] and Skyler Cranmer[a]

[a]Department of Political Science, The Ohio State University, 154 N Oval Mall, 43210, OH, US, [b]Department of Psychiatry, University of Pittsburgh School of Medicine, 3811 O'Hara St, 15213, PA, USA and [c]Department of Psychology and Center for Neural Science, New York University, 19 W 4th Street, 10003, NY, USA

[1]Seo-Eun Yang and James D. Wilson contributed equally to the preparation and writing of this manuscript.

[*]To whom correspondence should be addressed: wilsonj41@upmc.edu

FOR PUBLISHER ONLY Received on Date Month Year; accepted on Date Month Year

Abstract

Emerging research has begun investigating the neural underpinnings of the biological and psychological differences that drive political ideology, attitudes, and actions. Here we explore the neurological roots of politics through conducting a large sample, whole-brain analysis of functional connectivity (FC) across common fMRI tasks. Using convolutional neural networks, we develop predictive models of ideology using FC from fMRI scans for nine standard task-based settings in a novel cohort of healthy adults (n = 174, age range: 18-40, mean = 21.43) from the Ohio State University Wellbeing Project. Our analyses suggest that liberals and conservatives have noticeable and discriminative differences in functional connectivity that can be identified with high accuracy using contemporary artificial intelligence methods and that such analyses complement contemporary models relying on socio-economic and survey-based responses. Functional connectivity signatures from retrieval, empathy, and monetary reward tasks are identified as important and powerful predictors of conservatism, and activations of the amygdala, inferior frontal gyrus, and hippocampus are most strongly associated with political affiliation. Although the direction of causality is unclear, this study suggests that the biological and neurological roots of political behavior run much deeper than previously thought.

Key words: convolutional neural networks, deep learning, functional magnetic resonance imaging, political neuroscience

Significance Statement

Using state-of-the-art artificial intelligence techniques, we find that functional connectivity of the brain is highly predictive of one's political orientation. In the largest neuropolitics study to date, we find that of nine common tasks, functional connectivity from reward, retrieval, and empathy tasks were most predictive of political affiliation. We construct a powerful predictor of political ideology that enhances the use of common socio-demographic predictors. We identify regions of the brain are most influential in the prediction of liberalism and conservatism, possibly identifying the political brain. Although future research is needed to investigate how physical brain connections influence the relationship between functional connectivity and ideology, this study suggests that the neurological roots of political behavior run much deeper than previously thought.

Introduction

An individual's political ideology, which is a "set of beliefs about the proper order of society and how it can be achieved," (18; 6; 34) provides them with a framework by which to understand politics and make choices on complex issues. While political ideology is nuanced and multidimensional (9; 34; 51), it is frequently projected onto a single left-right dimension reflecting a continuum between liberalism and conservatism. The distribution of preferences across this continuum in a population often guides social, economic, and environmental policies thereby affecting many elements of society in democracies (49; 15; 43). As such, the underlying structure and determinants of liberal-conservative ideology is a major subject of investigation. Decades of research by political scientists have identified demographic patterns associated with political orientation (16; 48; 29; 54; 28) and documented how

© The Author 2021. Published by Oxford University Press. All rights reserved. For permissions, please e-mail: journals.permissions@oup.com

political orientation affects people's behavior (23; 1; 17; 19; 63). A nascent literature has started to probe the roots that ideology may have in the brain itself. This field, commonly referred to as political neuroscience, investigates the neural underpinnings of the biological and psychological differences that drive political ideology, attitudes, and actions (31). While much of traditional political science focuses on understanding politics at the aggregate level using experiments, surveys, and observational data, this new field uses technologies such as functional magnetic resonance imaging (fMRI) or event-related potential (ERP) to understand political attitudes/behavior at the individual level. The last decade has seen a handful of political neuroscience studies (2; 5; 31; 36; 38; 45; 46; 50; 66), which have suggested that there exist differences in the fundamental cognitive and emotional processes between liberals and conservatives. We substantially build on this past work by assessing how and to what extent functional connectivity networks differ systematically between liberals and conservatives using whole-brain analyses across nine distinct tasks on a novel and large dataset of 174 healthy young adults from The Ohio State University Wellbeing project (24). While we do not aim for a comprehensive review of the political neuroscience literature (see (31) for such a review), it is important to understand how our current study builds upon those in the literature and where it differs from them, answering several questions that remain open in the literature. Many studies of political orientation focus on how liberals and conservatives respond differently to task-specific stimuli that are specifically designed to activate processes related to political ideology (see for example, (5; 32; 44)). Others use emotional stimuli that, while not political, might activate processes correlated with political orientation (see for example (2)).The investigation of intrinsic functional connectivity patterns of the liberal and conservative brains absent any stimulus or with stimuli unlikely to activate political processes remains largely unexplored. To better understand the intrinsic neural differences of political ideology, we build on these past studies by conducting a whole-brain analysis using resting-state scans as well as a collection of scans from commonly used fMRI tasks. Although the tasks we consider were not designed to stimulate political attitudes, we seek to understand whether intrinsic signatures of political ideology are present and identifiable in off-the-shelf fMRI tasks.

A majority of political neuroscience studies predict ideology using the full time series, including responses and stimuli, of task-oriented BOLD responses (2; 5; 36; 38; 45; 50). While these analyses have done much to identify how particular regions of the brain interact with political stimuli, our study differs substantially in that functional connectivity analysis models the brain as a complex, networked system whose region-to-region relationships give rise to emergent political attitudes and behavior. Functional connectivity analysis can characterize how these region-to-region co-activations associate with political behavior and may reveal the subnetwork of brain regions that underlies the political brain. Finally, existing functional connectivity analyses (10; 47; 36) have investigated the effects of a single task or stimuli in isolation thereby leaving a substantial gap in formally comparing associations across tasks.

Examinations of functional connectivity patterns with subjects at rest found that political liberalism is associated with tighter communication between the dorsal anterior cingulate cortex – responsible for emotional processing – and the right insula – responsible for conflict monitoring (10; 47). In recent work, (36) found that connectivity between the orbitofrontal cortex (OFC) and precuneus as well as between the insula and frontal pole/OFC were particularly prominent in conservatives under stimuli designed to evoke anxiety. (2; 42) found that the function of the right amygdala, hippocampus, inferior part of the opercular frontal gyrus (IFGoperc), and the anterior cingulate cortex (ACG) are tied to political conservatism; the right IFGoperc is involved in risk aversion (42), and the ACG is associated with political liberalism. Our whole-brain analysis complements these previous findings by investigating whether, and to what extent, each region of the brain and their interactions plays a role in political attitude across common fMRI tasks.

Using the largest sample to date, we conducted a whole-brain analysis of functional connectivity across eight tasks and resting state to investigate four important and complementary questions about the neurological roots of politics: (i) to what extent can functional connectivity predict ideology? (ii) which task setting(s) from a collection of commonly used tasks are most suitable for the prediction of political ideology? (iii) to what extent does integrating functional connectivity predictors enhance the predictive ability of well-established survey-based political indicators? and (iv) which brain region(s) contribute most to the prediction of political ideology? To investigate these four questions, we employed a state-of-the-art deep learning technique known as BrainNetCNN (33) to analyze the associations of functional connectivity (FC) signatures across eight tasks and resting state with self-reported political ideology. Our analysis reveals that functional connectivity provides noticeable and discriminative features among liberals and conservatives, and that these patterns can be identified with high accuracy using contemporary artificial intelligence methods. We identify a collection of common fMRI tasks from which functional connectivity provides powerful predictive models of political ideology, and for each task, we characterize what brain regions are most strongly associated with liberalism and conservatism. Our analyses provide, for the first time, a systematic overview of the neural mechanisms of political ideology across a range of tasks and identifies which tasks and brain regions are related to political behavior for healthy adults.

Material and Methods

Participants and Imaging Data Acquisition

We use brain imaging data collected from 174 typically developing young adults from the Ohio State University Wellbeing project (age 18-40, mean 21.4; 61 males and 113 females) (24). Each participant underwent 1.5 hours of functional MRI recording, which consisted of eight tasks and resting-state scans using a 12-channel head coil on a Siemens 3T Trio MRI system with TIM, housed in the Center for Cognitive and Behavioral Brain Imaging at the Ohio State University (OSU). The eight tasks aim to observe subjects' brain activity involved in emotional picture viewing, emotional face viewing, episodic memory encoding and episodic memory retrieval, Go/No-go, monetary incentive, working memory, and a theory of mind task (see Table 1 for descriptions). We note that we use short hand naming conventions for these fMRI tasks throughout the remainder of the manuscript (provided in parentheses in Table 1) based on the intent or original use of the task as described in the reference from which that task was first designed. Please refer to Table 1 for the complete description of the task when evaluating the analytical results in this study. The 174 participants that we analyze are a subset of the 250

participants enrolled in the Wellbeing project; a subject was excluded if, during any of the tasks, part of the cerebral cortex was out of the field of view due to head motion (24). Scanning parameters, temporal resolutions, and task-based stimuli are described in detail in (24).

Self-reported survey data

Participants were also provided a series of survey-based questions, including questions regarding age, gender, their education and income, the education and income of their parents, the conservatism of their parents, as well as the conservatism of the city that they grew up in and the city they now live. With the exception of age and gender, these survey questions were answered on Likert scales. We use these covariates to build predictive models for political ideology as a benchmark against which we assess the utility of functional connectivity for predicting ideology. The outcome measure we consider is a subject's self-reported ideological position on a six point Likert scale from Very liberal to Very conservative. We provide descriptive summaries of these variables in Table 2, including the correlation of each feature with the Likert scale value of political ideology. We provide the survey questions and their possible answers in the supplementary material.

Preprocessing of Imaging Data

The fMRI data were first preprocessed using the minimal preprocessing Human Connectome Project pipeline (25). In particular, functional brain images were realigned to compensate for head motion, spatially smoothed (2-mm FWHM Gaussian kernel), and normalized with a global mean. The functional images were next coregistered to the T1-weighted structural images, normalized to the standard brain, and further refined using nonlinear registration in FSL (FMRIB software library, version 5.0.8). Minimal spatial smoothing was performed in volume space. Next, brain images were parceled into 269 regions of interest (ROIs) using the Automated Anatomical Labeling atlas (52). We excluded the 252nd ROI because it had all missing values in the time series of averaged BOLD signals, resulting in a 268 × 268 symmetric matrix. We recorded BOLD functional activation when subjects were in resting state and performed eight emotional and cognitive tasks. The task-associated BOLD activations were regressed out from the time series before connectivity analysis. We constructed functional connectivity networks by creating matrices where each row and column represent an ROI and the value of the (i, j)th entry of the matrix is the correlation coefficient between the ith and jth brain region from the time series of the averaged BOLD signals. We created these matrices for each participant and each task so that there are nine matrices per participant, one matrix for each of the nine tasks. Each functional connectivity matrix is represented as a full symmetric matrix with zeros along the diagonal and off-diagonal terms are between -1 and 1.

Statistical Analyses

We developed predictive FC models using the convolutional neural network (CNN) framework BrainNetCNN, which has architctures tailored to the brain proposed by (33). Convolutional neural networks (CNNs) have been increasingly successful not only in image classification and object recognition (39; 58) but also neuroscience to classify mental or physical disorder such as schizophrenia (35; 11; 64; 62), autism (26; 27), depression (7), stages of Alzheimer's disease (57), and infants born preterm (33). BrainNetCNN introduces two new layer types designed to capture topological locality in the brain. BrainNetCNN consists of both edge-to-node (E2N) layers and node-to-graph (N2G) layers that contain multiple convolutional filters layers of a particular shape. We use the Euclidean loss function as the final activation function in BrainNetCNN for regression and use very leaky rectified linear units (leaky-ReLU) as activation function within each layer in our model, where very leaky-ReLU function f is defined as $f(x) = 1(x < 0)(x/3) + 1(x \geq 0)(x)$. A mini-batch normalization is applied to each matrix before running the method. We use dropout with a rate of 0.5 after the N2G layer. We set the optimized hyperparameter in each fold via random search method by minimizing the training loss defined as the Euclidean loss between the predicted and true scores over the network parameters.

For each task t and individual k, BrainNetCNN yields an out-of-sample political ideology score $y_{t,k}$ – a continuous valued prediction for the self-reported (true) political ideology of individual k. We note that $y_{t,k}$ is the prediction of y_k from 10-fold cross validation when individual k is in the test set to prevent data leakage. Pearson correlation coefficients were used to asses relationships of these predictions with the true political ideology and a chi-squared test was used to evaluate significance of the correlation. Principal component analysis was applied to the matrix containing political ideology scores to determine the dimension of the scores. Principal component analysis was implemented using the *prcomp* function in R.

We next investigated the extent to which ideology scores could be used classify true ideology, when the true ideology was treated as a dichotomous outcome characterizing "conservative" (very conservative, somewhat conservative, conservative) and "liberal" (very liberal, somewhat liberal, liberal). We treat the ideology scores from BrainNetCNN as independent variables for individual i for which we fit a logistic regression model to predict that individual's dichotomized political ideology. These FC models were compared against other logistic regression models containing established survey-based responses, including variables describing age, sex, education, income, local leanings (including where an individual grew up and where they live now), and a subject's parents' political ideology (20; 13; 8; 59; 40) (survey-based predictors are summarized in Supplementary Table 1), as well as models containing both survey-based responses and FC. Monte Carlo cross validation with 1000 samples was used to compare all models. Test sets for each sample were chosen at random and contained a randomly chosen proportion of the total population of observations, with proportions between 0.05 and 0.50.

We next explored which connections learned by BrainNetCNN were most predictive of political ideology scores. To do so, we applied the deconvolutional network architecture from (56), which reconstructs brain connectivity within a deep learning architecture. This method computes the gradient of the score with respect to every input edge (i, j) of a functional connectivity matrix. In particular, the method calculates the partial derivative of $y_{t,k}$, $\frac{\partial y_{t,k}}{\partial E_{ij}}$, for every input edge E_{ij} of a functional connectivity matrix $\mathbf{E}$, $i, j = 1, \ldots, 268$. Edges with large-magnitude partial derivatives are edges that have a noticeable influence on the class score and thereby on a class posterior. The ROIs corresponding to such edges have greater importance in the prediction of political ideology. After averaging the partial derivative matrices over the entire dataset

Task	Description
Emotional pictures (Affect)	Subjects see photographs of the screen, one at a time. These photographs appear to the left or right of the center of the screen. The task is to indicate whether the picture is shifted to the left or right relative to green dot in the center of the screen. (2)
Emotional faces (Empathy)	Subjects are presented with male and female faces, one at a time. The task is to determine whether the faces are male or female. There are task conditions for neutral, happy, sad, and fearful faces (12).
Episodic Memory (Encoding)	Subjects see name and face pairings on a screen. The task is to decide whether the name goes well with the face on a 1-4 (poor to well) scale. There are 4 face conditions: young and old faces that are novel or have been repeated during the experiment (41).
Episodic Memory (Retrieval)	Subjects are asked to remember which names were paired with which faces from the episodic memory encoding task. The task is to indicate whether the face name pairs are the same from the previous task, completely novel, or if the face is repeated, but was not paired with the given name (41).
Go/No-Go	Subjects look images of single letters. They are asked to press a button when the letter is in the set A,B,C,D,E and not to press the button when the letter is in the set X,Y,Z (55).
Monetary Incentive Delay (Reward)	Subjects are asked to press a button as quickly as possible when a white square (cue) appears on the screen. Participants either win or lose money based on when and how fast they push the button (37).
Working Memory	Subjects are presented with a sequence of letters and switch between two memory tasks. In the rst, subjects are asked to indicate whether the current letter is underlined. In the second, subjects are asked to indicate whether the current letter is the same as or dierent from the one that was presented two letters ago (61).
Theory of Mind (ToM)	Subjects are presented with stories and true false statements about the stories. The task is to indicate whether the statement was true or false (14).
Resting State	Subjects are asked to close eyes, feel relaxed but stay awake.

Table 1. Descriptions of the tasks involved in the Wellbeing data set. Much of the text is reproduced from Supplemental Table S1 of the Wellbeing analysis in (24). In parentheses, we provide conventional names for the tasks that we will use throughout the remainder of the manuscript based on the original reference for each task given in the Description.

Demographic Characteristic	Summary	Correlation	p-value
Male, n (%)	61 (35.1)	0.180	**0.017**
Age, range, median, mean(sd)	18 - 40, 20, 21.43(3.83)	-0.133	0.080
	**# Likert responses (1/2/3/4/5/6)**		
Education	4/131/12/8/19	-0.137	0.071
Father Education	32/20/60/8/54	0.113	0.136
Mother Education	29/25/76/13/31	0.081	0.285
Father Conservatism	5/28/33/41/37/30	0.349	**$<$ 0.0001**
Mother Conservatism	9/37/46/35/31/16	0.417	**$<$ 0.0001**
Income	164/9/1/0/0/0	- 0.051	0.500
Parent's Income	20/22/35/29/39/29	0.194	**0.010**
Religiosity	44/40/24/29/26/11	0.279	**0.0001**
Origin City Conservatism	1/17/44/57/40/15	0.054	0.472
Current City Conservatism	16/64/64/23/6/1	-0.007	0.931
Conservatism	24/52/49/36/9/4	1.000	0

Table 2. Descriptive analysis of features describing the 174 individuals in the Well-being data set. Shown are summaries of each of the features used in the study as well as the correlation of the feature with the extremity of self-identified conservatism. The survey questions as well as possible answers (in Likert scales) are provided in the Supplemental material section S1.

and summing its magnitude over all the rows, we have the weighted degree centrality of brain regions as a measure of importance for the prediction. Then, 268 ROIs with the same anatomical structures in the AAL atlas are averaged to create the final 78 brain regions that were evaluated in our analyses.

To further test the importance of functional connectivity on predicting political ideology in the context of survey responses, we evaluated the variable importance of the FC predictors and survey predictors using an L1-penalized logistic regression model (65). The L1-penalized logistic regression model is a variable selection method that shrinks a subset of predictor coefficients to zero, thereby leaving important variables in the model. The absolute value of the estimated predictor's coefficient characterizes that predictor's importance in the model (60; 65). The L1-penalized model was tuned using cross-validation in the *glmnet* package in R software.

Results

We first investigated the pairwise associations of the FC political ideology scores obtained by BrainNetCNN, the

underlying dimension of these scores, as well as their associations with the true ideology of each participant. We next compared predictive models containing FC predictors from each task and survey-based responses using Monte Carlo cross validation. We assessed the importance of each task on the prediction of political ideology using variable importance measures from a L1-penalized logistic regression model. Finally, we applied deconvolutional network techniques to the FC predictive models to identify what brain regions were most predictive of political liberalism and conservatism. Details of these results are described in below.

	% Contribution				
Task	PC1	PC2	PC3	PC4	PC5
Affect	13.6	4.8	14.8	0.3	0.0
Empathy	12.9	5.2	0.1	10.0	15.9
Reward	11.5	2.9	1.0	6.9	31.3
Retrieval	10.4	0.2	45.7	3.0	4.6
Resting	7.9	20.2	27.2	15.8	1.2
GoNoGo	11.6	8.1	0.1	33.7	2.8
Encoding	9.8	38.0	4.9	0.2	1.2
Theory of Mind	10.8	7.3	4.9	20.8	30.7
Working Memory	11.5	13.2	1.3	9.3	12.2

Table 3. Variable contributions to the top five principal components of task political ideology scores.

Associations of political ideology with each fMRI task

BrainNetCNN provides raw political ideology scores for each fMRI task through a supervised learning task aimed at predicting the true political ideology of each individual. These ideology scores, their pairwise relationships, as well as their relationship with true political ideology is shown in Figure 1. Figure 1 highlights the associations of each task with overall ideology, as well as their associations with extreme and moderate ideologies. Particular attention was paid to associations with moderate (somewhat liberal, liberal, conservative, and somewhat conservative) and extreme views (very liberal and very conservative). Notably, the ideology scores from the affect, empathy, reward, and GoNoGo tasks were strongly associated with overall ideology (p-values < 0.001 independently and Bonferroni adjusted p-values < 0.05). When broken down by extremity, we found that only the reward task was statistically associated with extreme political views (correlation $= 0.750$, Bonferroni adjusted p-value < 0.05), and only the empathy task was statistically associated with moderate political ideology (correlation $= 0.342$, Bonferroni adjusted p-value < 0.05).

All tasks' ideology scores were statistically correlated with one another in overall ideology prediction, and all but one pair of tasks (empathy and retrieval) were strongly correlated across their moderate ideology predictions. To better understand these associations, we investigated the dimension of the political ideology scores by applying principal component analysis on the columns of the 174×9 ideology score matrix containing the political ideology scores for each participant for all 9 tasks. The scree plot and the biplot for the first two principal components are shown Figure 2, and the contributions of each fMRI task on the top five principal components are provided in Table 3. Principal component analysis suggests that the nine tasks can be well explained by five independent dimensions; all nine tasks contribute equally to the direction of most variation in the ideology scores, and only a subset of the tasks explain the remaining variability in prediction. In particular, we found that 42.5% of the total variability in the ideology score matrix was explained by the first principal component and that each task contributed approximately equally to this first component (range of 7.9 - 13.6 % contribution). The top five principal components explain roughly 80% of the total variability, suggesting that the dimension of these tasks is approximately five. Principal components 2 through 5 are each largely dictated by two or three tasks (PC2: resting and encoding; PC3: retrieval and resting; PC4: GoNoGo and theory of mind; PC5: empathy, reward, and theory of mind).

Predictive Capabilities of Functional Connectivity across Tasks and Resting State

A substantial body of work suggests that parental ideology is a particularly strong predictor of an individual's ideology (3; 4; 21; 30; 40). Research going back four decades shows parent's ideology to be a major determinant of an individual's ideology (1), and recent work using twin studies has shown that this relationship is likely partially heritable (4). Therefore, to evaluate the predictive performance of FC, we compare each model to a benchmark Parent Conservatism model, which contains the self-reported mother and father's conservatism. The mean and standard deviation of the area under the curve (AUC) metric for the test set from each sample in our cross validation study is reported in the left panel of Figure 3. We find strong and consistent predictive performance across the affect, retrieval, empathy, and reward connectivity networks - each of these models obtained predictive AUCs (mean AUC range: 0.625 - 0.674) that were statistically indistinguishable from the Parent Conservatism model (mean AUC $= 0.726$). This is significant because it shows that functional connectivity can provide at least as much information about an individual's liberalism/conservatism as the strongest predictor generally applied in political science research, and this finding speaks directly to the neurological roots of political ideology. Although the performances of the affect and empathy tasks are supported in past studies – classification of human faces into happy, sad, and fearful elicits emotions that may be related to political orientation (2) – the consistent results using connectivity from the reward task is novel and compelling. Despite the systematic literature for a possibility of the neural substrates of political behavior in reward-related processes, only (53) has studied the relationship of a reward-based task, the Risky-Gains task, and political ideology. Our finding complements this previous finding by mapping a monetary reward task to the brain activation in political orientation. Models that that contained all survey-based predictors and those that integrated FC predictors from each task with survey-based predictors obtained AUCs that were statistically higher than the Parent Conservatism model. The model that contained all survey-based predictors and FC variables was the most predictive model in the study (mean AUC $= 0.835$, sd $= 0.034$).

Variable Importance of FC Tasks and Survey-based Responses on Predicting Political Ideology

We fit L1-penalized logistic regression model to the full model containing all FC and survey-based predictors and report the

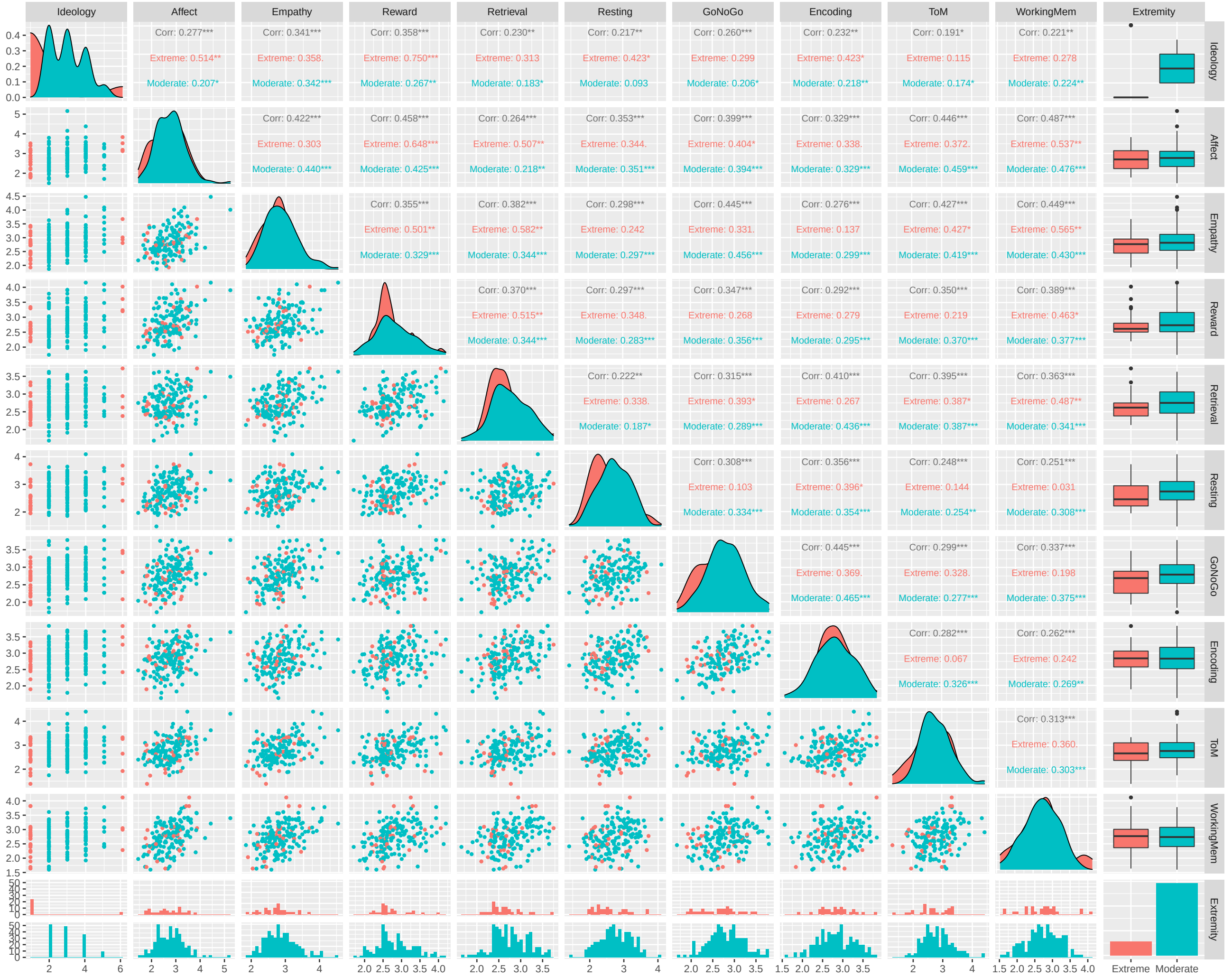

Fig. 1. Pairwise associations of FC scores and their associations with Political Ideology. Scatterplots show the relationship between the predicted political ideology score from each FC task once applied to BrainNetCNN and the true ideology. Points are colored according to extremity of true ideology: red points show extreme views (very liberal or very conservative) and blue points show moderate views (liberal, somewhat liberal, moderate, somewhat conservative, conservative). Correlations are provided for all ideology values (in black), moderate only (in blue), and extreme values (in red). Correlation values with *** are statistically significant with p-value < 0.001 and those with ** are statistically significant with p-value < 0.01 and those with * are significant at p-value < 0.05.

variable importance values for variables that had non-zero coefficients in Table 4. We take a strategy closely related to the data-driven approach followed by (66) in that we determine variable importance among task and survey-based responses through the use of predictive modeling and cross validation. Contrary to (66), however, we determine variable importance using the variable selection property of the LASSO (60) whose model was chosen by cross validation rather than by identifying variables with significant coefficients within the top 5% of predictive models over cross validation as done in that work. In line with past studies of parental influence on one's own political views, we found that parental variables (mother and father's education, mother and father's conservatism) were important in predicting political ideology. Three of the nine task-based FC variables were important in predicting ideology. The empathy, retrieval, and reward tasks were the most important variables in the model, surpassing the influence of parental and self survey-based variables. Taken together, our model and variable importance analyses identify three standard fMRI tasks from which FC strongly associates with political liberalism and conservatism.

Regional FC Associations with Political Ideology

We developed predictive FC models using the convolutional neural network (CNN) framework BrainNetCNN, which has architectures tailored to the brain proposed by (33). For each task, we first regressed out BOLD signals associated with stimuli in the task and then analyzed the FC matrix that models the co-activations of regions in the brain for that task. Features derived from each FC matrix from BrainNetCNN were then incorporated in logistic regression models to predict political ideology. These FC models were compared against other logistic regression models containing established survey-based responses, including variables describing age, sex, education, income, local leanings (including where an individual grew up and where they live now), and a subject's

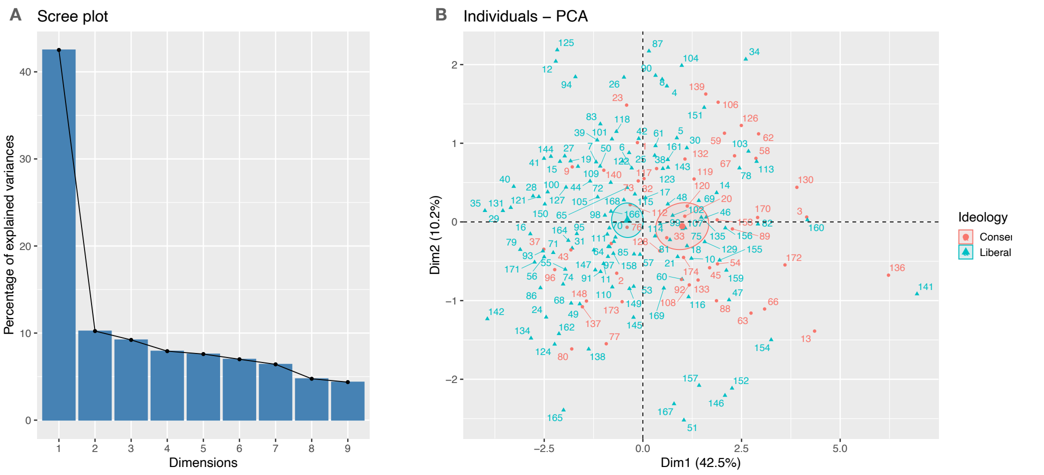

Fig. 2. Panel **A** shows a scree plot describing the percentage of variation explained by the principal components for the political ideology scores across all nine fMRI tasks. Panel **B** provides a biplot representing the principal component scores for all participants in the study. Points are colored according political ideology. Ellipses represent the 95% confidence interval of principal component scores for each ideology.

Variable	Importance	Coefficient (s.e.)	p-value
Empathy	0.570	0.151 (0.07)	**0.039**
Retrieval	0.460	0.148 (0.09)	0.087
Reward	0.371	0.103 (0.07)	0.142
Mother's Conservatism	0.340	0.082 (0.03)	**0.003**
Father's Conservatism	0.154	0.046 (0.03)	0.081
Mother's Education	0.083	0.039 (0.03)	0.172
Father's Education	0.053	0.029 (0.02)	0.253

Table 4. The variable importance values for an L1-penalized logistic regression that regresses political ideology (conservative or liberal) on the full model of all available survey-based predictors and FC predictors as well as the estimated effects and standard errors for each important variable from a logistic regression model. The L1-penalized model was tuned using cross-validation in the *glmnet* package in R software. Only variables that had non-zero importance in the resulting LASSO model are reported and variables are reported in order from greatest to least importance.

parents' political ideology (20; 13; 8; 59; 40) (survey-based predictors are summarized in Supplementary Table 1), as well as models containing both survey-based responses and FC. Monte Carlo cross validation with 100 samples was used to compare all models.

To better understand the biological mechanisms of political conservatism, we next investigate which brain regions are most predictive of political orientation across tasks using the deconvolutional network technique (DNT) from (56). The DNT technique quantifies the degree to which an edge between two regions is associated with prediction of political ideology, and is computed as the average partial derivative of the predicted political ideology with respect to the input edge. To identify the most predictive brain regions, we calculated the sum of the partial derivative magnitudes of all neighboring edges of each brain region. Regions with large values had, on average, connections that were most influential to the prediction of conservatism. The region-to-region associations with each political dimension for the three most important tasks - empathy, reward, and retrieval - are shown in Figure 4. The highest contributing edge for predicting conservatism and liberalism is given in Table 5.

**Task**	**Liberalism (ROI pair)**		**Conservatism (ROI pair)**	
**Affect**	MTG-R	PCG-L	SPG-R	SOG-L
**Empathy**	ORBmid-R	ITG-R	SFGdor-R	ROL-L
**Encoding**	ORBmid-R	LING-L	ORBmid-R	MTG-R
**GoNogo**	ITG-R	DCG-L	SMA-R	ITG-R
**Resting State**	SFGdor-R	ORBinf-R	SPG-R	SOG-L
**Retrieval**	ITG-R	ANG-L	INS-R	ANG-R
**Reward**	INS-R	CAU-R	ITG-R	SOG-L
**ToM**	SMA-R	LING-R	SPG-R	LING-L
**Working Memory**	SPG-R	ROL-R	INS-R	MTG-R

Table 5. Most influential regions for predicting political affiliation. For each task, the pair of regions that contributed most to the prediction of liberalism (left) and to the prediction of conservatism (right) are shown. Regions were identified using a deconvolutional network approach based on the neural network trained on functional connectivity for each task in the political ideology prediction task. Nodes are labeled by the following acronyms: IOG - inferior occipital gyrus; CUN - cuneus; ACG - anterior cingulate; AMYG - amygdala; SOG - superior occipital gyrus; ORB - orbital gyrus; IFGoperc - inferior part of the opercular frontal gyrus; ITG - inferior temporal gyrus; PCL - Paracentral lobule. A full list of regions in the AAL atlas are provided in supplemental material Table S2.

The DNT revealed that the left hippocampus (HIP), the left middle part of the orbital frontal gyrus, and the right amygdala (AMYG) are the most predictive of political ideology in the three most predictive tasks - reward, retrieval, and empathy tasks. The left middle part of the orbital frontal gyrus is a particularly strong predictor in all three of the tasks. Influential regions from the retrieval and empathy task show significant overlap with those identified by the reward tasks, including the

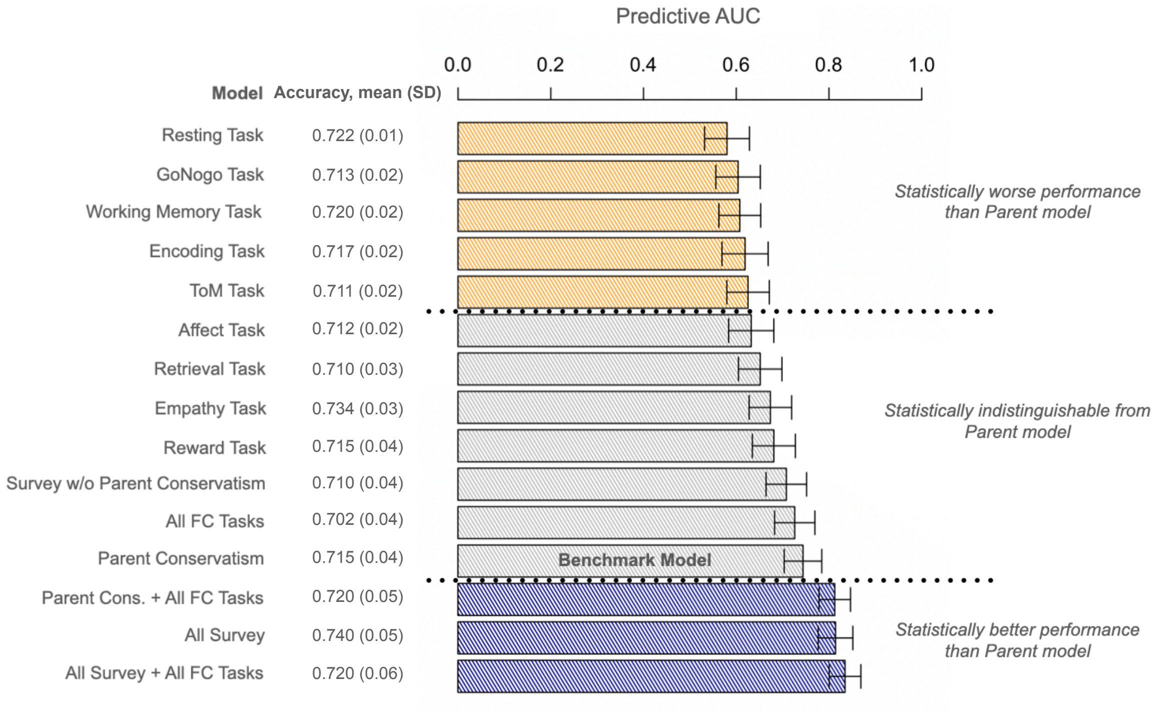

Fig. 3. Prediction performance of FC and survey-based predictive models. The accuracy and area under the curve (AUC) metric was calculated for each model using Monte Carlo cross-validation where the test set was a random sample with a random proportion of observations over 1000 samples. The length of each bar in the plot represents the mean predictive AUC, and error bars represent 95% confidence intervals. Bars are colored according to their performance when compared to the Parent Conservatism benchmark model containing mother and father conservatism as predictors. Survey-based models included age, education, income, how conservative the town was where a subject grew up, how conservative the city is where the subject lives now, a subject's parents' income, and mother and father conservatism.

left and right amygdala, and the right anterior cingulate cortex (ACG); however, the influential regions in the reward task tends to be much more widely distributed. The right inferior gyrus, the right Cuneus, the left inferior occipital lobe, and the left and right inferior part of the opercular frontal gyrus (IFGoperc) are highly influential brain regions for political ideology prediction. These findings are consistent with previous work (2; 42) who identified relationships of the AMYG, HIP, IFGoperc, and ACG with political ideology through task-based stimuli.

Discussion and Conclusion

Collectively, our analyses suggest that functional connectivity reveals noticeable and discriminative features among liberals and conservatives, and that these patterns can be identified with high accuracy using contemporary artificial intelligence methods. Our analyses provide a systematic overview of the functional mechanisms of political ideology across a range of tasks and identifies which tasks and brain regions are related to political behavior for healthy adults. We identified a subset of common fMRI tasks for which functional connectivity provides statistically indistinguishable predictions of political ideology as parental conservatism, and we found that functional connectivity signatures improve the predictive capability of models that utilize demographic and socio-economic indicators like age, education, geographic location, gender, conservative predispositions, and income. Indeed, the predictive model containing FC features in the study and all survey-based responses provided the strongest predictive capabilities of any model considered, giving an AUC boost of roughly 10% over a standard benchmark model using parental conservatism. From each task, we characterized what brain regions were most strongly associated with political ideology. These results were validated with cross-validation using the largest ever sample of subjects in a neuropolitics study.

While our analysis suggests that the empathy, reward, and retrieval tasks are the most strongly predictive of political attitude of the tasks we considered, we found that functional connectivity features from all of the tasks including resting state were correlated to political ideology (see Figure 1), suggesting that functional signatures of political ideology persist across tasks and resting state. Features from the reward task were the only to be statistically significantly associated to extreme political views. Although more work is needed to validate the relationship of reward decision-making with extreme political behavior, this finding is partly supported by (53) who found that the BOLD response of a Risky-Gains reward-based task was highly predictive of political orientation ($AUC = 0.829$), and is consistent with recent findings by (66) who found that reward sensitivity is implicated in ideological processes.

The empathy (emotional faces) task was the only task we found to be significantly correlated to moderate ideology. This may suggest that political thought may be closely tied to emotion and emotional response. This hypothesis is further supported by (2), who examined the relationships between the BOLD response from an that the emotional stimulus from images from a single disgusting image in an affect task and

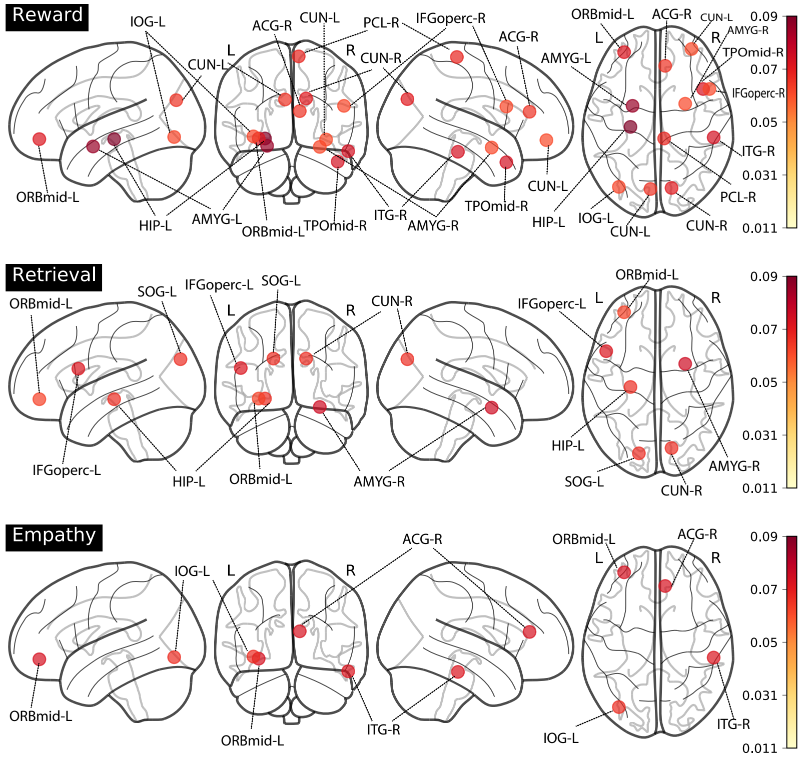

Fig. 4. Associations of brain regions with political ideology for the most three predictive tasks in the study. Highlighted regions were the most influential in predicting political ideology and had an importance score that was statistically higher than the average importance for the given task. Nodes are labeled by the following acronyms: IOG - inferior occipital gyrus; CUN - cuneus; ACG - anterior cingulate; AMYG - amygdala; SOG - superior occipital gyrus; ORB - orbital gyrus; IFGoperc - inferior part of the opercular frontal gyrus; ITG - inferior temporal gyrus; PCL - Paracentral lobule. A full list of regions in the AAL atlas are provided in supplemental material Table S2.

from multiple images with political ideology and found strong predictive performance (AUC = 0.845 and 0.981, respectively).

To date, political neuroscience has largely relied on the prediction of ideology using the full time series, including responses and stimuli, of task-oriented BOLD responses. There are two major differences that sets our current study apart. The first is that the current study is based on functional connectivity - that is, we analyze how the *relationships* between regions of the brain over each task predict political ideology. This is in contrast to analyzing the temporal trends in BOLD response of each regions as done in these past works. The second point is that we are concerned with functional signatures where task-related features dealing with reactions to task-based stimuli are regressed out before constructing functional connectivity matrices. Our study is the largest and most comprehensive study of functional connectivity for political behavior to date and complements previous task-based fMRI studies in political science by focusing on the identifications of functional connectivity signatures of political attitudes.

The most closely related study to our own was the recent work of (36), who investigated the relationship between functional connectivity of individuals undergoing anxious situations with political attitudes in South Korea. (36) examines psychological resilience and self-regulation as they pertain to "red" or "blue" brains, finding that conservatives tend to be more resilient and have better self-control, which helps them manage the fact that they are more sensitive to threat and anxiety. By contrast, we found that the connectivity of the right AMYG, HIP, IFGoperc, and ACG were most closely tied to political conservatism and that these findings were consistent across several tasks without political stimuli. Although more investigation is needed to characterize the ties of these brain regions and their connectivity with political ideology, it is interesting to note that these regions have been previously identified as important regions of interest in describing political behavior when activated by an emotional or political stimuli (2; 42).

The subjects in our data were scanned while performing a series of eight tasks, as well as in the resting state. None of these tasks were designed to elicit partisan responses and the resting state scan is particularly interesting because it allows us to test if brain connectivity can predict an individual's political orientation without any stimulus at all. Examining these distinct scans helps provide a general overview for the predictive ability of the tasks that distinctively capture brain regions related to political ideology. Our study was limited to nine fMRI tasks, but there are many other task-based settings that we did not consider that may provide additional insights into the neural signatures of political behavior. We look forward to that line of research.

Functional connectivity models the temporal coincidence of spatially distant neurophysiological events (22); however, FC does not imply any causal relationship between brain regions that exhibit these distant events. Indeed, correlated cross-regional activity may be mediated by additional structures or through other cortical–subcortical loops. As a result, strong

FC may be observed in absence of any structural connections, or may even be driven by external sources or due to effects of the experimental setup. We sought to minimize the effect of experiment in our analyses by regressing out task-associated BOLD activations from the time series for each task performed. We found that pairwise associations between the ideological scores of each pair of tasks were weak or moderate (see Figure 1), and that a majority of the variability in the 9 tasks could be explained by approximately five dimensions (Figure 2). Taken together, these results suggest that the predictive ability of each of the tasks was distinct and potentially complementary to one another. Our results offer exciting insights into understanding how the intrinsic activation of brain regions reflect differences in ideology; however, future research should investigate possible mediators to these functional relationships including physical white matter connections in the brain.

Our study is limited by the skew in political partisanship of the population. The number of conservative to liberal participants in the study was unbalanced (49 to 125), and the number of extreme conservatives considered in this study is small ($n = 4$). Our analysis, therefore, is limited in power by what can be said about differences in extreme political ideology. We dealt with this imbalance in an unbiased manner through cross validation, but we advocate for further study of the differences in political extremes in future research. The age of the participants was limited to adults (18 - 40). Therefore, our results may not hold in children and older adults (40+). Further investigation in these two groups is needed. Finally, our results are observational in the sense that the design of the experiments in the Wellbeing study were not political stimuli nor were participants randomized according to political ideology. Although the direction of causality remains unclear – do people's brains reflect the political orientation they choose or do they choose their political orientation because of their functional brain structure – the evidence here motivates further scrutiny and followup analyses into the biological and neurological roots of political behavior.

Acknowledgments

The authors thank the anonymous reviewers and the associate editor for their valuable suggestions. Z.L.L. would like to acknowledge William Cunningham for designing the OSU Wellbeing project with him, and Xiangrui Li for collecting the data in that project.

Supplementary Material

Supplementary material is available at PNAS Nexus online.

Funding

This work is supported in part by funds from the National Science Foundation (NSF: SES-1514750, SES-1461493, DMS - 1830547) and the National Institutes of Health (DA043079-01A1).

Author contributions statement

S.E.Y., J.D.W, S.J.C., and Z.L.L. conceived the analyses, S.E.Y. and J.D.W conducted the analyses, S.E.Y., J.D.W, S.J.C., and Z.L.L curated the data, S.E.Y., and J.D.W. wrote the manuscript, and S.E.Y., J.D.W, S.J.C., and Z.L.L reviewed and edited the manuscript.

Data availability

The anonymized functional connectivity data used for this analysis, as well as all code for the analysis, figures, and tables in the manuscript are publicly available at `https://github.com/jdwilson4/thePoliticalBrain`.

References

1. Christopher H Achen. Mass political attitudes and the survey response. *American Political Science Review*, 69(4):1218–1231, 1975.
2. Woo-Young Ahn, Kenneth T Kishida, Xiaosi Gu, Terry Lohrenz, Ann Harvey, John R Alford, Kevin B Smith, Gideon Yaffe, John R Hibbing, Peter Dayan, et al. Nonpolitical images evoke neural predictors of political ideology. *Current Biology*, 24(22):2693–2699, 2014.
3. John R Alford, Carolyn L Funk, and John R Hibbing. Are political orientations genetically transmitted? *American political science review*, pages 153–167, 2005.
4. John R Alford, Peter K Hatemi, John R Hibbing, Nicholas G Martin, and Lindon J Eaves. The politics of mate choice. *The Journal of Politics*, 73(2):362–379, 2011.
5. David M Amodio, John T Jost, Sarah L Master, and Cindy M Yee. Neurocognitive correlates of liberalism and conservatism. *Nature neuroscience*, 10(10):1246–1247, 2007.
6. Angus Campbell, Philip E Converse, Warren E Miller, and Donald E Stokes. *The american voter.* University of Chicago Press, 1980.
7. Bokai Cao, Lei Zheng, Chenwei Zhang, Philip S Yu, Andrea Piscitello, John Zulueta, Olu Ajilore, Kelly Ryan, and Alex D Leow. Deepmood: modeling mobile phone typing dynamics for mood detection. In *Proceedings of the 23rd ACM SIGKDD International Conference on Knowledge Discovery and Data Mining*, pages 747–755, 2017.
8. Ben Clements. Religion and ideology. In *Religion and public opinion in Britain*, pages 80–126. Springer, 2015.
9. Pamela Johnston Conover and Stanley Feldman. The origins and meaning of liberal/conservative self-identifications. *American Journal of Political Science*, pages 617–645, 1981.
10. C. L. Cox, J. T. Jost, F. X. Castellanos, M. P. Milham, C. Kelly, and J. J. Van Bavel. *Individual differences in political orientation are reflected in the intrinsic functional organization of the human brain.* Poster presented at the 5th Annual Meeting of the Social and Affective Neuroscience Society, New York, NY, 2012.
11. Jumana Dakka, Pouya Bashivan, Mina Gheiratmand, Irina Rish, Shantenu Jha, and Russell Greiner. Learning neural markers of schizophrenia disorder using recurrent neural networks. *arXiv preprint arXiv:1712.00512*, 2017.
12. Jean Decety, Stephanie Echols, and Joshua Correll. The blame game: the effect of responsibility and social stigma on empathy for pain. *Journal of cognitive neuroscience*, 22(5):985–997, 2010.
13. Tracy A Dennis, David M Amodio, and Laura J O'toole. Associations between parental ideology and neural sensitivity to cognitive conflict in children. *Social neuroscience*, 10(2):206–217, 2015.

14. David Dodell-Feder, Jorie Koster-Hale, Marina Bedny, and Rebecca Saxe. fmri item analysis in a theory of mind task. *Neuroimage*, 55(2):705–712, 2011.
15. Riley E Dunlap and Araon M McCright. A widening gap: Republican and democratic views on climate change. *Environment: Science and Policy for Sustainable Development*, 50(5):26–35, 2008.
16. Jon Elster. *Explaining social behavior: More nuts and bolts for the social sciences.* Cambridge University Press, 2015.
17. Robert S Erikson. The src panel data and mass political attitudes. *British Journal of Political Science*, 9(1):89–114, 1979.
18. Robert S Erikson and Kent L Tedin. *American public opinion: Its origins, content and impact.* Routledge, 2015.
19. Stanley Feldman. Measuring issue preferences: The problem of response instability. *Political Analysis*, 1:25–60, 1989.
20. Stanley Feldman and Christopher Johnston. Understanding the determinants of political ideology: Implications of structural complexity. *Political Psychology*, 35(3):337–358, 2014.
21. R Chris Fraley, Brian N Griffin, Jay Belsky, and Glenn I Roisman. Developmental antecedents of political ideology: A longitudinal investigation from birth to age 18 years. *Psychological science*, 23(11):1425–1431, 2012.
22. Karl J Friston. Functional and effective connectivity in neuroimaging: a synthesis. *Human brain mapping*, 2(1-2):56–78, 1994.
23. James K Galbraith. Mccarty, n., poole, kt, and rosenthal, h.: Polarized america. the dance of ideology and unequal riches, 2008.
24. Garren Gaut, Brandon Turner, Zhong-Lin Lu, Xiangrui Li, William A Cunningham, and Mark Steyvers. Predicting task and subject differences with functional connectivity and blood-oxygen-level-dependent variability. *Brain connectivity*, 9(6):451–463, 2019.
25. Matthew F Glasser, Stamatios N Sotiropoulos, J Anthony Wilson, Timothy S Coalson, Bruce Fischl, Jesper L Andersson, Junqian Xu, Saad Jbabdi, Matthew Webster, Jonathan R Polimeni, et al. The minimal preprocessing pipelines for the human connectome project. *Neuroimage*, 80:105–124, 2013.
26. Xinyu Guo, Kelli C Dominick, Ali A Minai, Hailong Li, Craig A Erickson, and Long J Lu. Diagnosing autism spectrum disorder from brain resting-state functional connectivity patterns using a deep neural network with a novel feature selection method. *Frontiers in neuroscience*, 11:460, 2017.
27. Anibal Sólon Heinsfeld, Alexandre Rosa Franco, R Cameron Craddock, Augusto Buchweitz, and Felipe Meneguzzi. Identification of autism spectrum disorder using deep learning and the abide dataset. *NeuroImage: Clinical*, 17:16–23, 2018.
28. Allyson L Holbrook. Attitude change experiments in political science. *Cambridge handbook of experimental political science*, 1:141–154, 2011.
29. Leonie Huddy, David O Sears, and Jack S Levy. *The Oxford handbook of political psychology.* Oxford University Press, 2013.
30. M Kent Jennings and Gregory B Markus. Partisan orientations over the long haul: Results from the three-wave political socialization panel study. *The American Political Science Review*, pages 1000–1018, 1984.
31. John T Jost, H Hannah Nam, David M Amodio, and Jay J Van Bavel. Political neuroscience: The beginning of a beautiful friendship. *Political Psychology*, 35:3–42, 2014.
32. Ryota Kanai, Tom Feilden, Colin Firth, and Geraint Rees. Political orientations are correlated with brain structure in young adults. *Current biology*, 21(8):677–680, 2011.
33. Jeremy Kawahara, Colin J Brown, Steven P Miller, Brian G Booth, Vann Chau, Ruth E Grunau, Jill G Zwicker, and Ghassan Hamarneh. Brainnetcnn: Convolutional neural networks for brain networks; towards predicting neurodevelopment. *NeuroImage*, 146:1038–1049, 2017.
34. Fred Nichols Kerlinger. *Liberalism and conservatism: The nature and structure of social attitudes*, volume 1. Lawrence Erlbaum Assoc Incorporated, 1984.
35. Junghoe Kim, Vince D Calhoun, Eunsoo Shim, and Jong-Hwan Lee. Deep neural network with weight sparsity control and pre-training extracts hierarchical features and enhances classification performance: Evidence from whole-brain resting-state functional connectivity patterns of schizophrenia. *Neuroimage*, 124:127–146, 2016.
36. Taekwan Kim, Ji-Won Hur, Seoyeon Kwak, Dayk Jang, Sang-Hun Lee, and Jun Soo Kwon. Intrinsic functional connectivity of blue and red brains: neurobiological evidence of different stress resilience between political attitudes. *Scientific reports*, 10(1):1–10, 2020.
37. Brian Knutson, Charles M Adams, Grace W Fong, and Daniel Hommer. Anticipation of increasing monetary reward selectively recruits nucleus accumbens. *Journal of Neuroscience*, 21(16):RC159–RC159, 2001.
38. Sekoul Krastev, Joseph T McGuire, Denver McNeney, Joseph W Kable, Dietlind Stolle, Elisabeth Gidengil, and Lesley K Fellows. Do political and economic choices rely on common neural substrates? a systematic review of the emerging neuropolitics literature. *Frontiers in psychology*, 7:264, 2016.
39. Alex Krizhevsky, Ilya Sutskever, and Geoffrey E Hinton. Imagenet classification with deep convolutional neural networks. In *Advances in neural information processing systems*, pages 1097–1105, 2012.
40. Jeffrey Lyons. The family and partisan socialization in red and blue america. *Political Psychology*, 38(2):297–312, 2017.
41. Anat Maril, Jon S Simons, Jason P Mitchell, Bennett L Schwartz, and Daniel L Schacter. Feeling-of-knowing in episodic memory: an event-related fmri study. *Neuroimage*, 18(4):827–836, 2003.
42. Mario F Mendez. A neurology of the conservative-liberal dimension of political ideology. *The Journal of neuropsychiatry and clinical neurosciences*, 29(2):86–94, 2017.
43. Gary Miller and Norman Schofield. The transformation of the republican and democratic party coalitions in the us. *Perspectives on Politics*, 6(3):433–450, 2008.
44. Mark Mills, Frank J Gonzalez, Karl Giuseffi, Benjamin Sievert, Kevin B Smith, John R Hibbing, and Michael D Dodd. Political conservatism predicts asymmetries in emotional scene memory. *Behavioural Brain Research*, 306:84–90, 2016.
45. H Hannah Nam, John T Jost, Lisa Kaggen, Daniel Campbell-Meiklejohn, and Jay J Van Bavel. Amygdala structure and the tendency to regard the social system as legitimate and desirable. *Nature Human Behaviour*, 2(2):133–138, 2018.

46. H Hannah Nam, John T Jost, Michael R Meager, and Jay J Van Bavel. Toward a neuropsychology of political orientation: exploring ideology in patients with frontal and midbrain lesions. *Philosophical Transactions of the Royal Society B*, 376(1822):20200137, 2021.
47. Roger Newman-Norlund, Jessica Burch, and Katie Becofsky. Human mirror neuron system (hmns) specific differences in resting-state functional connectivity in self-reported democrats and republicans: A pilot study. *Journal of Behavioral and Brain Science*, 2013, 2013.
48. Pippa Norris, Robert E Goodin, et al. *Oxford Handbook of Political Behavior*. Oxford Handbooks of Political, 2007.
49. Christina Pagel, David W Bates, Don Goldmann, and Christopher F Koller. A way forward for bipartisan health reform? democrat and republican state legislator priorities for the goals of health policy, 2017.
50. Walker S Pedersen, L Tugan Muftuler, and Christine L Larson. Conservatism and the neural circuitry of threat: economic conservatism predicts greater amygdala–bnst connectivity during periods of threat vs safety. *Social cognitive and affective neuroscience*, 13(1):43–51, 2018.
51. Mark A Peffley and Jon Hurwitz. A hierarchical model of attitude constraint. *American Journal of Political Science*, pages 871–890, 1985.
52. Edmund T Rolls, Marc Joliot, and Nathalie Tzourio-Mazoyer. Implementation of a new parcellation of the orbitofrontal cortex in the automated anatomical labeling atlas. *Neuroimage*, 122:1–5, 2015.
53. Darren Schreiber, Greg Fonzo, Alan N Simmons, Christopher T Dawes, Taru Flagan, James H Fowler, and Martin P Paulus. Red brain, blue brain: Evaluative processes differ in democrats and republicans. *PLoS one*, 8(2):e52970, 2013.
54. Shalom Schwartz. A theory of cultural value orientations: Explication and applications. *Comparative sociology*, 5(2-3):137–182, 2006.
55. Daniel J Simmonds, James J Pekar, and Stewart H Mostofsky. Meta-analysis of go/no-go tasks demonstrating that fmri activation associated with response inhibition is task-dependent. *Neuropsychologia*, 46(1):224–232, 2008.
56. Karen Simonyan, Andrea Vedaldi, and Andrew Zisserman. Deep inside convolutional networks: Visualising image classification models and saliency maps. *arXiv preprint arXiv:1312.6034*, 2013.
57. Heung-Il Suk, Seong-Whan Lee, Dinggang Shen, Alzheimer's Disease Neuroimaging Initiative, et al. Hierarchical feature representation and multimodal fusion with deep learning for ad/mci diagnosis. *NeuroImage*, 101:569–582, 2014.
58. Christian Szegedy, Wei Liu, Yangqing Jia, Pierre Sermanet, Scott Reed, Dragomir Anguelov, Dumitru Erhan, Vincent Vanhoucke, and Andrew Rabinovich. Going deeper with convolutions. In *Proceedings of the IEEE conference on computer vision and pattern recognition*, pages 1–9, 2015.
59. Chris Tausanovitch. Income, ideology, and representation. *RSF: The Russell Sage Foundation Journal of the Social Sciences*, 2(7):33–50, 2016.
60. Robert Tibshirani. Regression shrinkage and selection via the lasso. *Journal of the Royal Statistical Society: Series B (Methodological)*, 58(1):267–288, 1996.
61. L Xu, SC Fagan, and JL Waller. Edwards d, borlongan cv, zheng j, hill wd, feuerstein g, hess dc. low dose intravenous minocycline is neuroprotective after middle cerebral artery occlusionreperfusion in rats. *BMC Neurol*, 4(7), 2004.
62. Weizheng Yan, Sergey Plis, Vince D Calhoun, Shengfeng Liu, Rongtao Jiang, Tian-Zi Jiang, and Jing Sui. Discriminating schizophrenia from normal controls using resting state functional network connectivity: A deep neural network and layer-wise relevance propagation method. In *2017 IEEE 27th international workshop on machine learning for signal processing (MLSP)*, pages 1–6. IEEE, 2017.
63. John R Zaller et al. *The nature and origins of mass opinion*. Cambridge university press, 1992.
64. Ling-Li Zeng, Huaning Wang, Panpan Hu, Bo Yang, Weidan Pu, Hui Shen, Xingui Chen, Zhening Liu, Hong Yin, Qingrong Tan, et al. Multi-site diagnostic classification of schizophrenia using discriminant deep learning with functional connectivity mri. *EBioMedicine*, 30:74–85, 2018.
65. Ji Zhu and Trevor Hastie. Classification of gene microarrays by penalized logistic regression. *Biostatistics*, 5(3):427–443, 2004.
66. Leor Zmigrod, Ian W Eisenberg, Patrick G Bissett, Trevor W Robbins, and Russell A Poldrack. The cognitive and perceptual correlates of ideological attitudes: a data-driven approach. *Philosophical Transactions of the Royal Society B*, 376(1822):20200424, 2021.
